# Assessing the Impact of Downsampled ECGs and Alternative Loss Functions in Multi-Label Classification of 12-Lead ECGs

**DOI:** 10.1007/s13239-025-00797-8

**Published:** 2025-09-17

**Authors:** Bjørn-Jostein Singstad, Eraraya Morenzo Muten

**Affiliations:** 1 Medical Technology and E-health, Akershus University Hospital, Lørenskog, Norway; 2 Department of Endocrinology, Obesity and Nutrition, Vestfold Hospital Trust, Tønsberg, Norway; 3 Department of Research and Innovation, Vestfold Hospital Trust, Tønsberg, Norway; 4 Institute of Clinical Medicine, University of Oslo, Oslo, Norway; 5 Department of Biomedical Engineering, Institut Teknologi Bandung, Bandung, Indonesia

**Keywords:** ECG, Convolutional neural network, Multi-label classification, Explainable AI, Double soft F1-loss

## Abstract

**Background:**

The electrocardiogram (ECG) is an almost universally accessible diagnostic tool for heart disease. An ECG is measured by using an electrocardiograph, and today’s electrocardiographs use built-in software to interpret the ECGs automatically after they are recorded. However, these algorithms exhibit limited performance, and therefore, clinicians usually have to manually interpret the ECG, regardless of whether an algorithm has interpreted it or not. Manual interpretation of the ECG can be time-consuming and requires specific skills. Therefore, better algorithms are clearly needed to make correct ECG interpretations more accessible and time-efficient. Algorithms based on artificial intelligence (AI) have demonstrated promising performance in various fields, including ECG interpretation, over the past few years and may represent an alternative to manual ECG interpretation by doctors.

**Results:**

We trained and validated a convolutional neural network with an Inception architecture on a dataset with 88253 12-lead ECGs, and classified 30 of the most frequent annotated cardiac conditions in the dataset. We assessed two different loss functions and different ECG sampling rates and the best-performing model used double soft F1-loss and ECGs downsampled to 75Hz. This model achieved an F1-score of $0.420\pm 0.017$, accuracy $= 0.954 \pm 0.002$, and an AUROC score of $0.832 \pm 0.019$. An aggregated saliency map, showing the global importance of all 12 ECG leads for the 30 cardiac conditions, was generated using Local Interpretable Model-Agnostic Explanations (LIME). The global saliency map showed that the Inception model paid the most attention to the limb leads and the augmented leads and less importance to the precordial leads.

**Conclusions:**

One of the more significant contributions that emerge from this study is the use of aggregated saliency maps to obtain global ECG lead importance for different cardiac conditions. In addition, we emphasized the relevance of evaluating different loss functions, and in this specific case, we found double soft F1-loss to be slightly better than binary cross-entropy (BCE). Finally, we found it somewhat surprising that drastic downsampling of the ECG led to higher performance than higher sampling frequencies, such as 500Hz. These findings contribute in several ways to our understanding of the artificial intelligence-based interpretation of ECGs, but further studies should be carried out to validate these findings in other datasets from other patient cohorts.

## Introduction

Cardiovascular disease (CVD) is one of the leading causes of death worldwide. Data from the World Health Organization estimate that 17.9 million people died from CVD in 2016, which represented $31\%$ of all global deaths that year [1]. Early detection of individuals at risk for CVD could potentially reduce the severity of the disease and the number of deaths caused by it.

Electrocardiography is a non-invasive and widely used method to record electrocardiograms (ECG), which has enabled clinicians to interpret, diagnose and prognosticate heart disease since the beginning of the 20th century [2]. An ECG is the result of a measurement of the electrical activity of the heart by recording the voltage potential from electrodes placed on the patient’s skin. Electrocardiography is generally easier to set up and more cost-effective compared to other diagnostic methods such as echocardiogram and magnetic resonance imaging of the heart. On the other hand, one of the challenges is that the ECG can be difficult to interpret correctly. Correct interpretation can be time-consuming and requires a high level of expertise [3].

In the 1950s, it became possible to convert analog ECG signals to a digital format and this led to the development of digital interpretation algorithms in the 1960s [4]. Today, most of the modern and clinically used electrocardiographs are equipped with built-in interpretation software. The software interprets the ECG and prints an interpretive text that may indicate different pathologies. Studies show that there are several limitations to the automatic interpretation algorithms [4, 5]. The errors, done by the automatic interpretation algorithms, imply that doctors have to read over the ECGs to ensure the predicted cardiac condition is correct. This is time-consuming for the doctors and leads to high interpretation variability. This emphasize that it is necessary to develop better algorithms for interpreting ECGs, as this may lead to less time-consuming interpretation for doctors, less interpretation variability, and better diagnostic performance, which may lead to earlier detection and treatment of CVD patients.

In the past decades, several new important trends have converged and may potentially be ushering in a new technological era with significance to ECG interpretation. Firstly, ECGs are now increasingly stored in digital format, allowing computerized analysis of massive data sets. Secondly, personal sensors such as training monitors and smartwatches (e.g., Apple Watch, Withings Watch, Samsung Galaxy Watch) now include ECG recording abilities, further expanding access to ECGs and the range of people studied. Finally, artificial intelligence (AI) or more specifically deep learning (DL) has shown remarkable abilities in classifying signal data [6], and more specifically also ECG data [7–13].

Despite the good performance of the DL-based ECG interpretation models, the doctors remain responsible for the diagnosis, and diagnostic models should then be considered as decision support tools, but the complexity in DL models can make the decision inaccessible to humans, often referred to as the black box phenomenon [14]. Trying to solve the black box phenomenon has led to the development of another sub-field within AI, eXplainable Artificial Intelligence (XAI) [15], to make the model’s decision more human-interpretable. XAI methods such as gradient activation map (Grad-CAM) [16], Local Interpretable Model-agnostic Explanation (LIME) [17] and SHAP [18] have already been used to calculate class activation maps for a given prediction, showing which part of the raw ECG waveform was most important for the DL model’s prediction. The majority of these studies have focused on local explanations (explaining the classification of one observation) and explaining single-label classification models [19–22], while only a few studies have used XAI on multi-label classification models [23, 24]. Van De Leur et al. 2020 showed how beneficial XAI can be in their study, where they discovered novel disease-specific ECG features in Phospholamban (PLN) mutation carriers [19]. For many other diseases, the DL model will likely rely on very subtle patterns and combinations of features from different leads, and even though these get highlighted and displayed to the doctors in the class activation map, it might be hard for them to understand or verify the relationship between the features used by the DL-model. Another way to benefit from XAI in ECG classification could potentially be to discover which leads are more important when classifying certain cardiac conditions.

In this paper, we extend upon the work of the George Moody Challenge 2020 and 2021, where the primary objective was to achieve a multi-label classification of cardiovascular cardiac conditions through the use of raw ECG waveform data, as reported in previous research [25, 26]. Our contribution to the 2020 edition of the challenge involved the implementation of convolutional neural networks (CNNs) with rule-based methodologies, as detailed in our earlier study [27]. Subsequently, during the 2021 challenge, we employed CNNs in classifier chains [28]. In this current investigation, we conduct a comparative analysis of Inception models that are trained with binary cross-entropy (BCE) and double soft F1-loss functions. Furthermore, we systematically examine how variations in ECG sampling frequency influence classification efficacy. In addition, we apply XAI techniques to discern which of the 12 ECG leads hold the greatest global significance across an extensive number of cardiac conditions. This manuscript introduces a deep learning paradigm utilizing a convolutional neural network (CNN) with an Inception-based architecture, well-suited for capturing multi-scale temporal features inherent in ECG signals. We then assess the proposed framework over a spectrum of ECG sampling rates to evaluate its adaptability to variations in signal resolution, an aspect that remains significantly underexplored in ECG analyses. The study also clarifies the differential impact of two prevalent loss functions: categorical cross-entropy and double soft F1-loss on classification outcomes, especially relevant in scenarios characterized by imbalanced datasets. The results obtained from our study provide valuable insights into the design of model architectures and the adoption of training strategies beneficial to the more effective classification of ECG signals.

## Methods

### Data

**Fig. 1:**
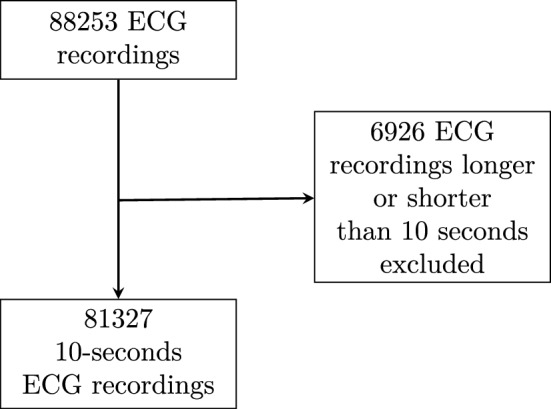
Patients with an ECG recording shorter or longer than 10 s were excluded from the development set. 6926 ECGs were excluded and the remaining 81327 ECGs were used to train and validate the model

We used ECG data from seven different open-access databases [25, 26, 29–33], with a total of 88253 12-lead ECGs in waveform format. All ECGs different from 10 sec in recording length, likely to be Holter-ECGs or Exercise Stress Test ECGs, were excluded, and the remaining 81327 10-second 12-lead ECGs were used for further development and validation as shown in Fig. 1. Using only 10-seconds recording lengths was advantageous for several reasons. Firstly, it is likely that all 10-second ECGs are resting ECGs as the 10-sec recording is the global standard for resting ECGs. Furthermore, this ensures a similar measurement protocol and thus a more homogeneous dataset. Secondly, 1D CNNs demonstrate optimal performance when utilizing a consistent input size. Given that 92% of the ECG recordings in our dataset were precisely 10 sec in duration, this configuration maximizes the dataset′s size without necessitating any cropping or padding adjustments to adapt the ECG signals to a fixed length of 10 sec. Cropping in particular could lead, unintentionally, to leaving out important information from the ECG.

Each ECG waveform was stored in .mat-files and each .mat-file had a corresponding .hea-file containing metadata such as the ECG recording length, sample frequency, the patient’s age, gender and annotated cardiac condition. There was a total of 133 different expert-annotated cardiac conditions in the dataset, but similar to the organizers of George Moody PhysioNet Challenge 2021, we choose to only consider 30 of the cardiac conditions. The selection of cardiac conditions for the George Moody PhysioNet Challenge 2021 by the organizers is justified based on their relative frequency, clinical importance, and electrophysiological characteristics, which enable precise diagnosis solely from ECG recordings [26]. Table 1, presents the prevalence rates for each of the 30 cardiac conditions, highlighting a significant variability in these rates. In some cases, the ECG has been annotated with more than one of the 30 cardiac conditions. This makes the current challenge a multi-label classification task with more than 3000 unique combinations of cardiac conditions among the patients in the dataset.

**Table 1:** The prevalence of the 30 classified cardiac conditions after excluding patients with an ECG recording length different from 10 s

Cardiac conditions	Prevalence
1st Degree AV Block	3291
Atrial Fibrillation	5062
Atrial Flutter	8509
Bradycardia	267
Bundle Branch Block	511
Complete Left Bundle Branch Block	218
Complete Right Bundle Branch Block	2015
Incomplete Right Bundle Branch Block	2306
Left Anterior Fascicular Block	2665
Left Axis Deviation	7952
Left Bundle Branch Block	1260
Low QRS Voltage	1765
Nonspecific Intraventricular Conduction Disorder	2101
Pacing Rhythm	1694
Poor R Wave Progression	656
Premature Atrial Contraction	2827
Premature Ventricular Contractions	1259
Prolonged PR Interval	393
Prolonged QT Interval	2152
Q Wave Abnormal	2261
Right Axis Deviation	1482
Right Bundle Branch Block	2331
Sinus Arrhythmia	4176
Sinus Bradycardia	19303
Sinus Rhythm	30426
Sinus Tachycardia	10261
Supraventricular Premature Beats	267
T Wave Abnormal	12673
T Wave Inversion	4340
Ventricular Premature Beats	731

### Preprocessing

#### ECG Processing

Approximately $85\%$ of the electrocardiograms (ECGs) within the development set were originally sampled at a frequency of 500 hertz (Hz). To ensure uniformity across all data, each ECG underwent resampling to achieve a consistent sample frequency prior to conducting training and validation procedures. In this investigation, we evaluated the model’s performance by systematically resampling the signals from its initial sampling frequency  to 500, 400, 300, 200, 100, 75, 50, and 25 Hz, and assessed the impact of such changes.

#### Label Processing

In this study, we employed one-hot encoding to transform all 30 cardiac conditions under investigation into a format suitable for analysis. Each electrocardiogram (ECG) recording was associated with a 30-bit array comprising binary values of ones and zeros. Within this array, a binary one indicated the presence of an annotation for the corresponding cardiac condition, demonstrating that the patient had been identified with this specific condition. Conversely, a binary zero signified the absence of an annotation, indicating that the patient had not been associated with that particular cardiac condition.

### CNN Architecture

**Fig. 2:**
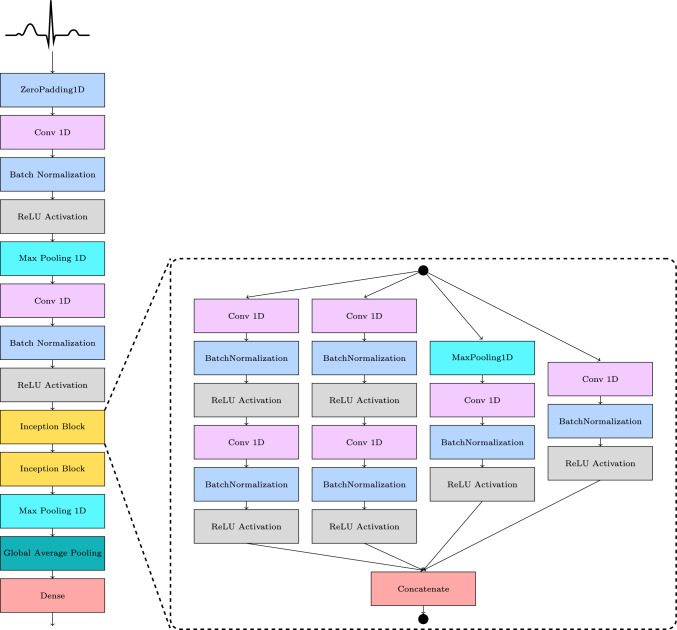
Inception model architecture. Each block represents a mathematical operation in the convolutional neural network. The blocks inside the dashed lines represent an Inception block

Building upon the framework provided by the two-dimensional Inception architecture [34], we developed a CNN model specifically tailored for the processing of multi-lead, one-dimensional time-series datasets, such as 12-lead ECGs. Moreover, this CNN is capable of predicting multi-label outputs. The model construction and development were executed using the Tensorflow framework [35] and the high-level model architecture is illustrated in Fig. 2.

The proposed model combines parallel convolutional pathways of different receptive fields to capture both short-term and slightly longer-term features efficiently. The model accepts an input tensor $\mathcal {X} \in \mathbb {R}^{b,l,c}$, where *b* is the batch size, *l* is the signal length and *c* is the number of channels. An initial zero padding layer adds symmetric padding of size 3 to preserve boundary information after the subsequent convolutional layers. This is followed by a convolutional layer with a kernel size of 7 and 64 filters, which captures long-range temporal patterns. This is followed by batch normalization and ReLU activation function to stabilize and introduce non-linearity, respectively. A max pooling layer with a pool size of 3 and stride of 2 reduces temporal resolution. Subsequently, a second Conv 1D layer with a kernel size of 5 and 64 filters is applied, again followed by batch normalization and ReLU activation.

#### Inception Modules

The core of the architecture comprises two stacked Inception blocks. Each Inception block is composed of four parallel convolutional sub-paths.

The first path applies a single convolution with kernel size 1 to extract point-wise features. The second path begins with a convolution with kernel size $=1$ followed by a convolution with kernel size $=3$ to capture medium-range temporal dependencies. The third path begins with a convolution with kernel size $=1$ followed by a $5 \times 5$ convolution to capture slightly longer-range temporal features than the second path. The fourth path applies max pooling, with kernel size $=3$ and stride $=1$, followed by a convolution with kernel size $=1$. Each path includes batch normalization and ReLU activations after each convolution. The outputs of all four branches are concatenated along the feature dimension, allowing the model to learn multi-scale representations.

After the Inception modules, a max pooling layer with a kernel size of 7 and stride of 2 further downsamples the features. A global average pooling layer reduces the tensor by one dimension and leaves a feature vector $\varvec{x} \in \mathbb {R}^{b,64}$. Subsequently, a fully connected dense output layer is employed with $\varvec{C}$ nodes, using sigmoid activation functions. Here, $\varvec{C}$ indicates the number of target classes, which is 30, allowing for multi-label classification.

### Loss Function

A loss function is used to compute the error of the prediction made by the model during the training phase. The computed errors are used to adjust the weight coefficient in the model using backpropagation [36]. In multi-label classification tasks, especially in clinical datasets such as ECGs, class imbalance is a major challenge. Some cardiac conditions appear very frequently (e.g., sinus rhythm), while others are rare (e.g., prolonged PR interval), which can lead to standard loss functions to prioritize the majority classes during training.

To address this, we propose the use of double soft F1-loss, a differentiable approximation of the F1-score that symmetrically penalizes both false positives and false negatives across all classes. A previous study claimed that different variations of F soft loss could be beneficial when performing multi-label classification with imbalanced classes [37]. Our contribution lies in applying and evaluating a double soft F1-loss in multi-label ECG classification tasks, a context where this formulation is both novel and practically impactful.

Unlike binary cross-entropy, which evaluates each class independently, double soft F1-loss treats the predictions in aggregate, capturing the dependencies introduced by label imbalance and overlap. This provides a more balanced optimization target, especially valuable when rare conditions carry high clinical importance.

The binary cross-entropy loss is defined as:

1 $$\begin{aligned} \mathcal {L} (y,\hat{y}) = - (y\log {\hat{y}} + (1-y)log(1-\hat{ y})) \end{aligned}$$

Equation 2 shows how double soft F1-loss ($\mathcal {L}_{F_1}$) is calculated. The small number ($10^{-16}$) is added to the denominator to prevent the function from dividing by zero.

The double soft F1-loss is calculated as:

$$\begin{aligned} & \text {tp} = \sum _{i=1}^n \hat{y}_i \cdot y_i \\ & \text {fp} = \sum _{i=1}^n \hat{y}_i \cdot (1-y_i)\\ & \text {fn} = \sum _{i=1}^n (1 - \hat{y}_i) \cdot (1-y_i) \end{aligned}$$

2 $$\begin{aligned} \mathcal {L}_{F_1} = 1 - \frac{2 \cdot \text {tp} }{2 \cdot \text {tp} + \text {fp} + \text {fn} +10^{-16}} \end{aligned}$$

This loss was minimized using the Adam optimizer [38]. Integrating this formulation into model training directly addresses the skewed label distribution in ECG datasets and leads to improved performance across both frequent and infrequent cardiac conditions.

### Training and Validation

The model was trained and evaluated on the dataset using 10-fold stratified cross-validation. The data were stratified based on the prevalence of the cardiac conditions to ensure a similar distribution of cardiac conditions in all 10 cross-validation folds. The models were trained for 15 epochs, with a batch size of 30 and a learning rate of 0.001.

The model performance was scored using the area under the receiver operating characteristic (AUROC) curve, F1-score and average accuracy across all classes (hereby just referred to as accuracy). Equation 3 shows how we compute accuracy by comparing the true label (*y*) and the predicted label ($\hat{y}$) for each ECG recording, $n_s$ and then finding the average accuracy for each class *c* and finally taking the average across all classes, $n_c$.

3 $$\begin{aligned} accuracy \;(y,\hat{y})=\frac{1}{n_{c}} \sum _{i=0}^{n_{c}-1} \frac{1}{n_{s}} \sum _{j=0}^{n_{s}-1} 1 \cdot (\hat{y}_{ij}=y_{ij}) \end{aligned}$$

### Explainability

**Fig. 3:**
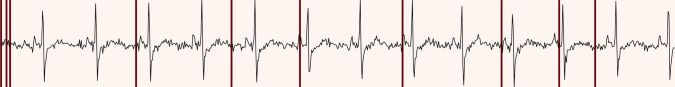
Lead aVL visualized together with its class activation map for a patient correctly classified with atrial fibrillation with a probability of 0.998. The red vertical lines show the features in the ECG that contributed most towards the prediction, according to the Local Interpretable Model-agnostic Explanation (LIME) model

To find the relative importance of the features in the ECGs, a LIME model was trained to fit the input data (ECGs) to the output predictions from the Inception model. A LIME model is a linear surrogate model that is easier to interpret compared to a deep neural network [17]. The LIME models purpose is to mimic the behaviour of the neural network and at the same time be interpretable. One LIME model was trained and tested for each of the 30 classes. As output, a LIME model provides a class activation map that has an equal shape to the input. Values close to zero in the class activation map is equivalent to low activation, while higher values mean higher activation. Figure 3 shows an example of one ECG lead (aVL), visualized in the same plot as the corresponding activation map for that specific lead. The LIME model has learned to quantify the contribution of each sample in the ECG in the atrial fibrillation classification performed by the Inception model. The dark red lines indicate high activation, while the brighter color indicates lower activation. This example is what is called a local explanation because it only explains the model’s behavior on data from a single observation, whereas global explanations are used to explain the feature importance and the model’s behavior on all observations in the dataset.

#### Developing the LIME Model

**Fig. 4:**
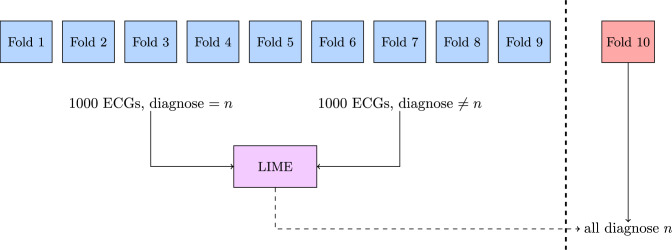
Training the Local Interpretable Model-Agnostic Explanation (LIME) model was done sampling data from the first 9 folds in the 10-fold cross-validation split. 1000 ECGs with the cardiac condition of interest and 1000 ECGs without the cardiac condition of interest were used in the training. The trained model was then applied on all ECGs with the cardiac condition of interest in the 10th fold

We trained a LIME model for each of the 30 classes using the training data from the 1-9th cross-validation split during the model development. For each LIME model, 1000 ECGs were randomly sampled with replacement from the 30 different classes, with 1000 ECGs labeled with the class of interest and 1000 ECGs labeled with classes different from the class of interest being used to train the LIME model. The trained LIME models were then applied to the ECGs in the 10th cross-validation split. The *n*-th LIME model was applied to all ECGs labeled with the *c*-th class in the validation split. Figure 4 shows the described flow in a graphical way.

## Results

### Loss Function

**Fig. 5:**
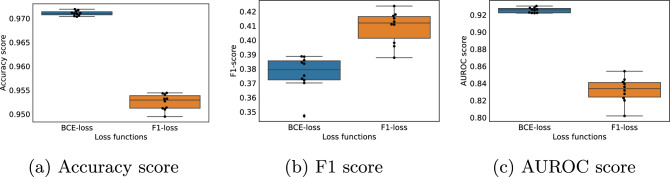
Classification performance achieved by the Inception model trained on 12-lead ECGs, using binary cross-entropy loss and double soft F1-loss. The box plots are accumulated scores obtained on the validation folds during 10-fold cross-validation, using ECGs resampled to 100Hz. The results are visualized in terms of accuracy (**a**), F1 (**b**) and AUROC scores (**c**)

The box plots illustrated in Fig. 5 present a comparative analysis of the Inception model’s performance when leveraging the double soft F1-loss as opposed to the BCE loss. Each individual box summarizes the results from ten cross-validation folds in terms of accuracy (Fig. 5b), F1-score(Fig. 5b), and AUROC score (Fig. 5c).

In our empirical analysis, we conducted a comparison between the double soft F1-loss and the BCE loss. The results of our evaluation indicate that, when both losses were applied to the Inception model, the model equipped with the BCE loss demonstrated superior performance in terms of accuracy and the Area Under the Receiver Operating Characteristic curve (AUROC) score. Conversely, the implementation of the double soft F1-loss resulted in the highest F1-score for the model. Given the highly imbalanced nature of the dataset employed in this study, we prioritized the F1-score as the primary criterion for selecting the appropriate loss function for subsequent experimental investigations. Based on this criterion, the double soft F1-loss was ultimately chosen, as it achieved the highest F1-score among the two options.

### Sampling Frequency

**Fig. 6:**
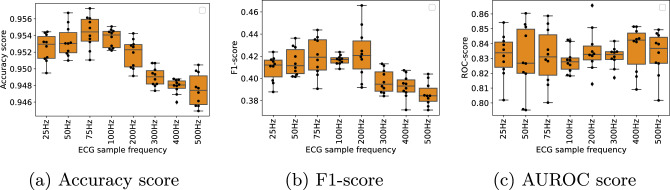
Classification results performed on the ECGs at different sampling frequencies. Each box represents the accumulated scores obtained on the validation folds by the Inception model, using double soft F1-loss, during 10-fold cross-validation. **a** shows the accuracy score, **b** shows the F1-score and **c** shows the AUROC scores

To evaluate the influence of ECG sampling frequency on classification performance, we generated eight distinct versions of the initial dataset by resampling it at varying sampling rates: 25 Hz, 50 Hz, 75 Hz, 100 Hz, 200 Hz, 300 Hz, 400 Hz, and 500Hz. Subsequently, we implemented eight separate Inception models, which were trained and validated using a 10-fold cross-validation approach. Each of these models incorporated the double soft F1-loss function and underwent training for a total of 15 epochs. The training process utilized a batch size of 30 and adopted the Adam optimizer with an initial learning rate set to 0.001. Figure 6 presents a comparative analysis of these eight models concerning their cross-validated accuracy, F1-score, and AUROC score.

### Explainability

**Fig. 7:**
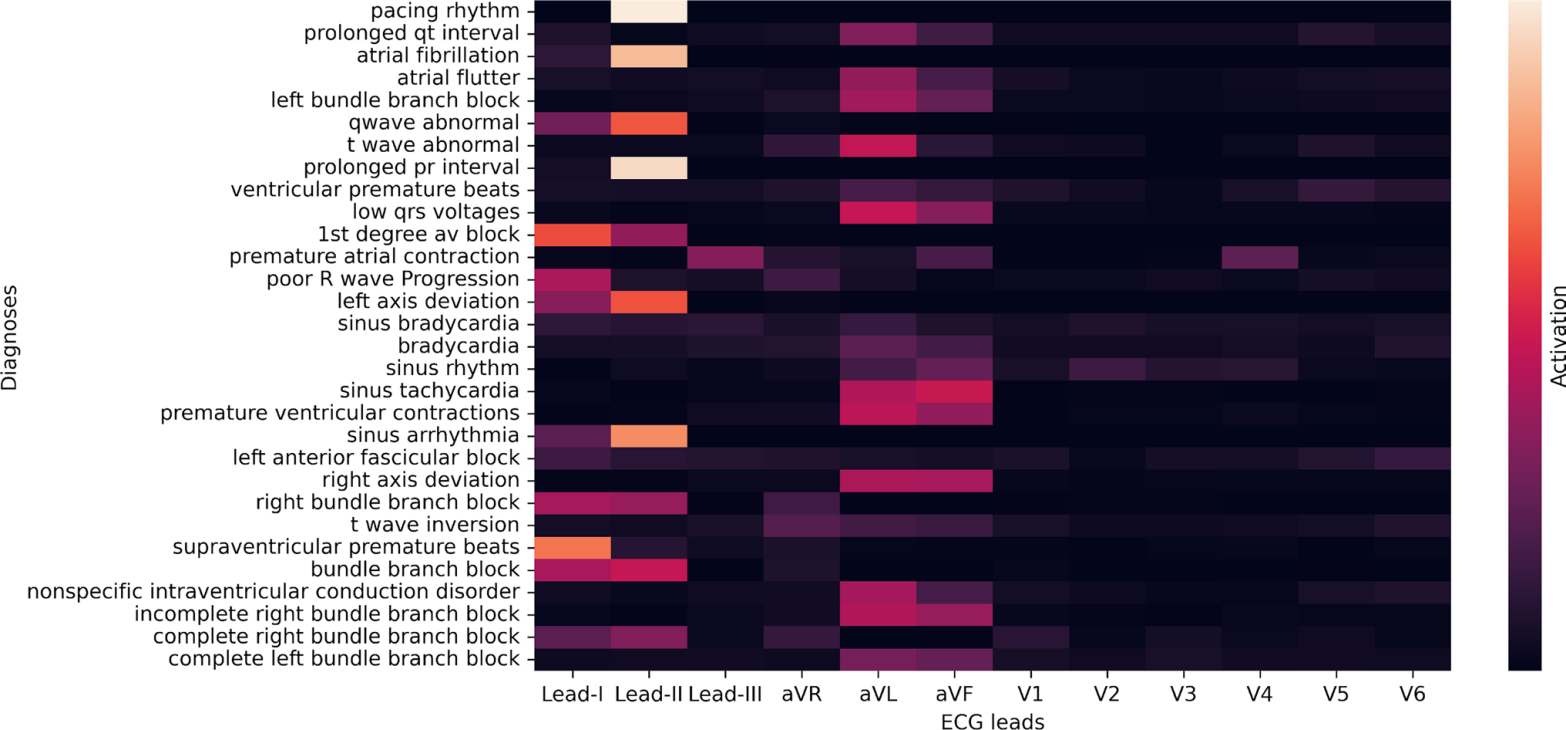
Aggregated results from the class activation maps provided by the LIME model when interpreting the predictions from the Inception model that is interpreting the validation data. The x-axis shows the 12 ECG leads and the y-axis represents the 30 different cardiac conditions represented in the dataset. Dark colors in the saliency map mean low activation, while bright mean high activation

Finally, an Inception model, with a double soft F1-score as the loss function, was trained on ECG signals resampled to 75Hz. The Inception model was trained on 9 out of the 10 folds defined by the cross-validation. The 10th fold was used as validation data. One LIME model was trained and tested for each of the 30 cardiac conditions considered in this study. Figure 7 shows a saliency map of the ECG leads with the overall highest activation for each of the 30 cardiac conditions. The saliency map was obtained by calculating the total activation for each lead in each ECG. This was done for each of the 30 cardiac conditions in the 10th validation fold.

## Discussion

This study demonstrates an Inception-type CNN trained to do multi-label classification using a dataset comprising 12-lead ECGs with an imbalanced distribution of labels. The Inception model using double soft F1-loss performed slightly better than the Inception model using BCE loss. As far as we know this is the first study that has reported results using double soft F1-loss for ECG classification. Secondly, we evaluated how resampling the ECG signals to various frequencies, specifically ranging from 25Hz to 400Hz, affected the performance of the classification. Lastly, we also employed an XAI technique called LIME in order to find the ECG lead with the highest class activation for each of the 30 cardiac conditions considered in this study. To the best of our knowledge, this is the first time class activation maps have been used to determine global ECG lead importance for different cardiac conditions.

During the development of the Inception model, we compared two different loss functions, BCE and double soft F1-loss. We found that double soft F1-loss gave a significantly better F1-score (Fig. 5b), which is considered to be the most important metric in a heavily imbalanced dataset such as the one used in this study. It is however somewhat surprising that the model using BCE loss achieved better accuracy and AUROC score than the model using double soft F1-loss. A plausible explanation seems to be that the BCE model performed well in classifying the negative cases, which was in majority for almost all cardiac conditions. This could give a high accuracy score, and the model using double soft F1-loss may have a more balanced performance on negative and positive cases, resulting in a higher F1-score.

The study revealed an unexpected outcome where the classification performance improved when ECG sample frequency was reduced from the original 500 Hz. As seen in Fig. 6 both the accuracy (Fig. 6a) and F1-score (Fig. 6b) performance reach their peak around 75Hz. Our findings are in contrast to another previous study showing a steep decrease in accuracy when using a sample frequency lower than 250 Hz when doing QRS detection using 1D CNNs. It should also be noted that the same study showed almost no increase in performance going from 250 Hz to 2000Hz. [39]. The observed increase in performance, using downsampled ECG signals, in our study could seem to be counterintuitive since one would expect the ECG to lose vital information when being downsampled. A possible explanation for the increase in performance at lower sample rates could be caused by an ideal ratio between the convolution kernel size and the ECG sample rate, in order to effectively detect pathologich features in the ECG. Even though we did some experiments increasing the kernel size at higher ECG sample frequencies, this did not give the same improvement in classification performance as lowering the ECG sampling frequency. However, more research is needed to conclude.

The saliency map in Fig. 7 is an aggregation of all class activation maps achieved from the 10th cross-validation fold. The figure reveals that the limb leads, specifically leads I, II, III, aVR, aVL, and aVF, demonstrate the most significant activation across various cardiac conditions. In contrast, the precordial leads, identified as V1 through V6, typically show minimal or no activation. This phenomenon might be attributed to the predominance of rhythm disorders in the dataset, which are more effectively observed via the limb leads. Therefore, during the phase in which the neural network is trained, we fundamentally assume that it modifies the coefficient weights related to the limb leads, resulting in an enhanced emphasis on these specific leads. Moreover, among the limb leads, lead II appears to hold the most significance. We propose that the rationale behind this observation lies in the comparatively high signal-to-noise ratio associated with lead II in contrast to other leads. The model, as a consequence, may have been trained to give precedence to information obtained from lead II due to its likelihood of being less contaminated by noise.

### Augmentation

Augmentation has shown promising effects in various image classification tasks [40]. Therefore we hypothesized that augmentation might have a good effect on signal and ECG classification as well. More specifically, we tried to add random noise and baseline wander to the ECG signals. However, these augmentations did not significantly improve the performance of our models.

The random noise was induced by adding a random number (*N*) in the range of the ± standard deviation ($\sigma$) of all values in the current ECG recording, shown in Eq. 4.

4 $$\begin{aligned} y_{i_n} = y_i + N(-\sigma ,\sigma ) \end{aligned}$$

Baseline wander was induced to the signal by adding a cosine wave from 0 to $2\pi$ and shifting the cosine wave randomly between 0 and $2\pi$. The amplitude of the signal, with n samples, was randomly set by multiplying a random number (N) drawn from the distribution of all values in an ECG recording, shown in Eq. 5.

5 $$\begin{aligned} y_{i_{bw}}=cos(2\pi \frac{y_i}{n}+N(0,2\pi ))\cdot N(-\sigma ,\sigma ), \; i=0,1,2..(n-1) \end{aligned}$$

**Fig. 8:**
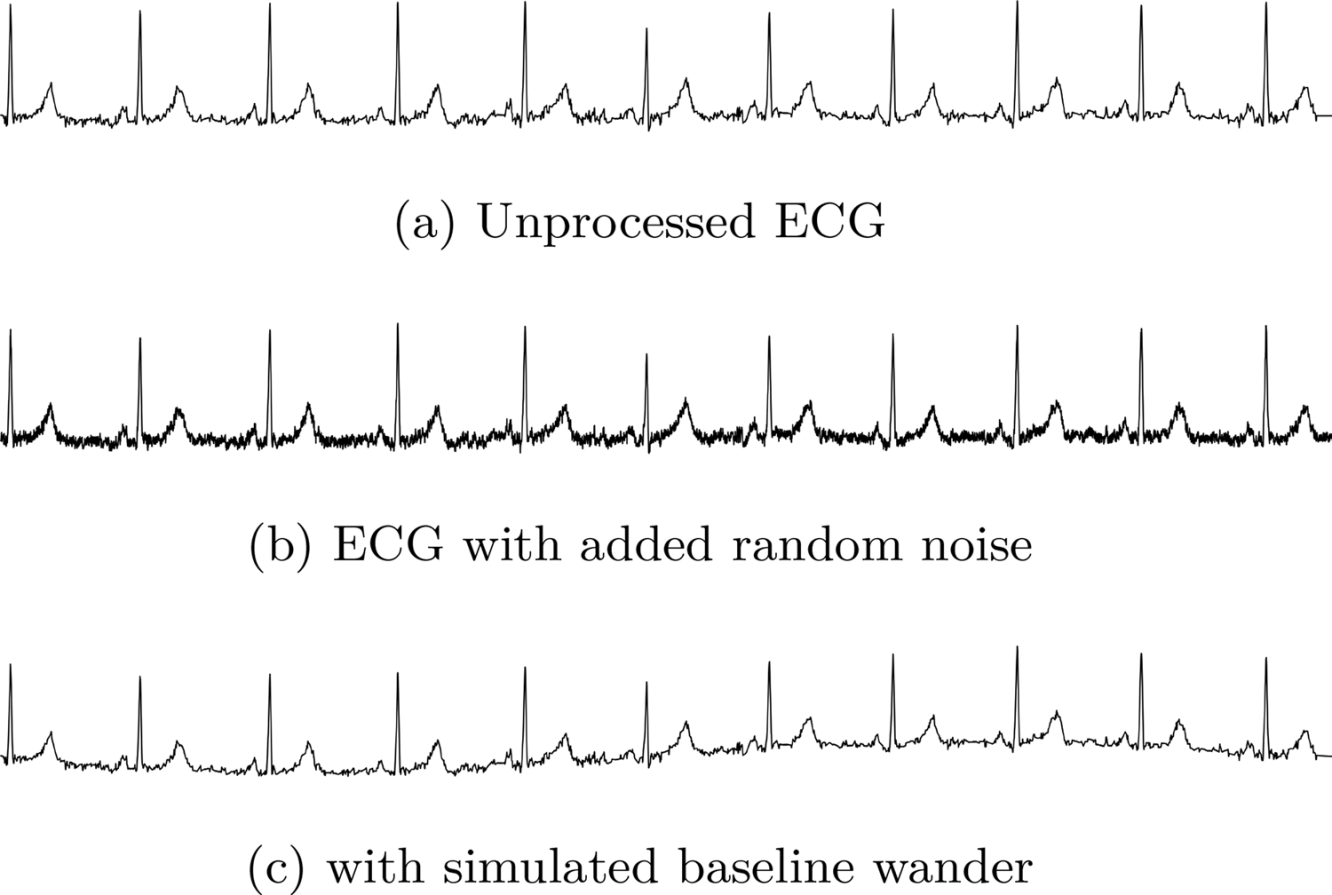
Comparing an unprocessed ECG (**a)** with the same ECG with added noise (**b**) and simulated baseline wander (**c**)

Figure 8a shows an example of an unprocessed ECG and Fig. 8b shows the same ECG with added random noise using the method described in Eq. 4. Figure 8c shows the ECG in Fig. 8a with simulated baseline wander as described in Eq. 5.

### Limitation

One key limitation of the results in this work is that we did not test the model on a separate and independent test set. The selection of optimal loss function and sampling rate could therefore have resulted in overfitting to the present dataset. However, the datasets used to train and validate the model are from different hospitals across the world and is therefore likely to represent a great diversity of patients, but anyway, validation on an external test set is needed to control the model for potential overfitting that could have occurred in our study.

In order to create the class activation maps and the aggregated lead importance diagram (Fig. 7) we used the LIME framework. One of the limitations of this approach is that the LIME module we used was originally intended to be used on recurrent neural networks and not CNNs, as done in this study. Secondly, previous research has stated that methods such as LIME are too generic and should be used with care on waveform data [41]. Comparing activation maps from LIME with model-specific explanation methods, such as Grad-CAM [16], would therefore be interesting.

### Future Perspectives

Future studies should consider loss functions other than binary cross-entropy when training neural network-based multi-label classification models on imbalanced datasets. We also suggest that future studies should explore novel model architectures or configurations to take better advantage of the subtle and detailed information of ECGs sampled at 500 Hz or higher.

For a more thorough understanding of model outputs, it is recommended that subsequent research projects incorporate the expertise of medical doctors or cardiologists in the validation process of ECG activation maps. This collaborative effort will facilitate a comprehensive evaluation of the effectiveness and practical applicability of XAI in this field.

## Conclusion

The primary aim of this study was to train a multi-label ECG classifier to achieve the best possible performance, given the unbalanced dataset. Furthermore, we used this model to obtain class activation maps and based on those we found the leads that were considered the most important to classify each of the 30 cardiac conditions. We also found that double soft F1-loss might improve the performance when classifying heavy imbalanced datasets. In addition, we observed that reducing the sampling frequency of the ECG from 500 Hz all the way down to 75 Hz increased the classification performance. Lastly, the combined feature maps derived from the XAI approach indicated a greater significance of the limb leads and augmented leads when compared to the precordial leads across all 30 classes. This observation is logical given the substantial occurrence of arrhythmic diagnoses within this dataset.

## Data Availability

The ECG datasets used in this study are publicly available and were sourced from multiple open-access repositories, including those referenced in the PhysioNet/Computing in Cardiology Challenge 2020 and 2021. Detailed references and links to the data sources can be found in the manuscript’s references [25, 26, 29–33].
